# A pilot study for an integrated diabetes screening, referral, and care program within a low-income community in Mexico

**DOI:** 10.3389/fcdhc.2025.1694026

**Published:** 2026-01-14

**Authors:** Ana Aurora Silva Baeza, Gabriel Q. Shaibi, Stephanie L. Ayers, María Velentina Toral Murillo, Christine Karkashian, Jesús A. Moya, Maria G. Zavala-Cerna

**Affiliations:** 1 Unidad Académica Ciencias de la Salud, Universidad Autónoma de Guadalajara, Zapopan, Jal, Mexico; 2 Center for Health Promotion and Disease Prevention, Edson College of Nursing and Health Innovation, Arizona State University, Phoenix, AZ, United States; 3 Southwest Interdisciplinary Research Center, School of Social Work, Arizona State University, Phoenix, AZ, United States; 4 Escuela de Psicología, Facultad de Ciencias de la Salud, Universidad Latina de Costa Rica, San José, Costa Rica; 5 Escuela de Medicina, Facultad de Ciencias de la Salud, Universidad Latina de Costa Rica, San José, Costa Rica

**Keywords:** community health workers, diabetes self-management education and support, glycemic control, social determinants of health, type 2 diabetes

## Abstract

**Objective:**

To determine the feasibility and acceptability of a coordinated community-based intervention for low-income adults with type 2 diabetes (T2D) that included (1) screening and referral, (2) shared decision-making (SDM), and (3) diabetes self-management education and support (DSMES).

**Methods:**

Participants were screened for T2D through a mobile health unit in a low-income community in Guadalajara, Jalisco, Mexico, and referred for follow-up in a primary care health center serving that community. Primary care physicians (PCPs) within the health center were trained on SDM for T2D, and community health workers (CHWs) were trained to deliver DSMES. Feasibility was measured by the number of community members screened and referred for care, the number of PCPs implementing SDM, and the number of CHWs hired and trained on DSMES. Acceptability was assessed by the percentage of participants who completed the 3-month DSMES program. Potential clinical impact was determined by effect sizes of changes in HbA1c between baseline and 3 months. Other measurements included waist circumference (WC), body weight, diabetes distress, and diabetes self-care activities, assessed at baseline, and at 1 and 3 months during the study period.

**Results:**

With respect to feasibility, all PCPs from the clinic completed the SDM training and were able to implement it in their primary practice. The DSMES training was completed by 4 (50%) of CHWs, and 3 were selected to deliver the course to study participants. Related to acceptability, 182 community members were screened, of which 42 were eligible for participation and 23 were successfully enrolled. Out of six programmed sessions, average participant attendance was 80% with 60.9% of participants retained at three months. Changes in HbA1c from baseline to 3 months were 10.1 ± 2.7 to 9.4 ± 3.1.

**Discussion:**

The use of community screening to refer low-income people living with T2D to a clinic-based SDM and DSMES intervention was feasible with large effect sizes for changes in HbA1c. The high attrition rates suggest that alternative strategies may be necessary to keep patients engaged in care.

## 1 Introduction

Type 2 diabetes (T2D) is a chronic disease associated with long-lasting multiorgan complications and is considered a global public health epidemic (1). In Mexico, the prevalence of T2D is 18.3% (2), and it is the leading cause of death in adults (3). It is estimated that the number of Mexicans living with T2D will almost double over the next two decades (4).

The increased T2D-related morbidity and mortality observed in Mexico, compared with other countries, may be related to limited access to medical care (5) and self-management education, coupled with a negative impact of social determinants of health (SDoH) as root causes for care disparities associated with T2D. SDoH are conditions in which people are born, live, and age and include income, educational attainment, limitations in access to health care, reliable transportation, stable housing, psycho-social resources and supports, as well as nutritious food security (6). Not only does T2D disproportionately impact low-income and minority populations (7), but the negative impacts of SDoH on T2D extend from individuals to communities through poverty, residential segregation, and crowded housing (8, 9).

Benefits from screening programs may involve early detection and referral to make lifestyle changes, which will delay the incidence of complications (10). Screening programs include questionnaires to score risk as well as blood-based biomarkers such as fasting plasma glucose (FPG) or hemoglobin A1c (HbA1c) (11). In community settings where SDoH are active ingredients, increasing access to point of care (POC) measures of HbA1c can facilitate the uptake of T2D screening (12) and identify more individuals at high risk for T2D (13, 14).

Interventions aimed at addressing T2D disparities must consider the various SDoH, as they represent root causes of poor T2D outcomes among populations at high risk (15). Such interventions should consider individual, social, community, and societal drivers as well as behavioral, cultural, and health system factors that can be leveraged to support improved T2D outcomes (16).

To this end, previous studies have been conducted to examine various education and decision-making modalities to improve glycemic control in patients with T2D. Studies have demonstrated that community health workers (CHWs) can provide diabetes self-management education and support (DSMES) among populations experiencing health disparities (17). In addition, shared decision-making (SDM) can be employed by health care providers to engage patients with T2D in identifying the best-fitting treatment options given the evidence as well as their values, preferences, and context (18). However, to date, there is no information related to the benefits of combining both CHWs delivering DSMES and primary care providers (PCPs) using SDM for glycemic control among patients with T2D.

To address these gaps, we conducted a pilot study with the primary aim of examining the feasibility and acceptability of an integrated community-based screening, referral, and intervention program in a low-income community. As a secondary aim, we examined changes in relevant T2D-related outcomes following the intervention.

## 2 Methods

### 2.1 Study design

This was a non-randomized pilot study conducted between 2023 and 2024 among inhabitants of a low-income community affected by SDoH in Guadalajara, Jal. Mexico. The study was reviewed and approved by the Institutional Review Board of UAG with number CEI/2023/002. Written informed consent was obtained prior to data collection.

### 2.2 Study population

The priority community is inhabited by ~27,600 people from ~6,560 households, where the average age is 32 years old, and the average schooling is 9 years. The community is located in an urban area with limited economic opportunities. Although housing quality allows for proper construction, usually houses accommodate more than one family. There are some areas with insufficient street lighting and electricity. The community has access to reliable potable water. However, residents face underemployment and rely heavily on the informal economy (street vending, construction work), which typically offers no labor benefits or social security. Income levels are low compared to other urban areas of the same metropolitan zone.

A mobile clinical unit was driven to churches or local markets to screen potential participants for elevated HbA1c using point of care (POC) testing with a DCA Vantage^®^ analyzer. Those with an HbA1c ≥ 6.5% were referred to the community clinic where the intervention would be delivered for follow-up care.

Within the clinic, subjects were invited to participate in the pilot study; upon acceptance, eligibility was assessed, questionnaires were administered, and study samples to assess HbA1c were collected. Inclusion criteria were age > 18 years old, residents of the priority community for at least 3 previous years, and HbA1c between 6.5% and 12%. Exclusion criteria included planning to move in the following year, severe T2D complications that would limit their participation in the study, severe anemia (Hb < 10), recent massive blood loss, or having a genetic condition with hemoglobin variants.

### 2.3 Study intervention

The intervention consisted of a combination of a culturally grounded DSMES delivered by trained CHWs and PCPs engaging with patients through diabetes-centered care discussions as part of SDM.

### 2.4 Study measurements and data collection

#### 2.4.1 Primary and secondary outcomes

Primary outcomes included feasibility and acceptability. Feasibility was measured by the number of trained CHWs on DSMES and the number of PCPs trained in SDM. Acceptability was measured by the number of participants enrolled in the intervention, the number of intervention sessions attended, and the number of participants who completed the intervention and follow-up data collection. As secondary outcomes, we explored the potential clinical impact of the intervention by examining changes in HbA1c from baseline to post-intervention (3 months). Other measurements included waist circumference (WC), body weight, diabetes distress, and diabetes self-care activities, assessed at baseline and 1 and 3 months after enrollment.

#### 2.4.2 Clinical assessment

Height was assessed without shoes using a wall-mounted stadiometer to the nearest 0.1 cm, weight was assessed in light clothing using a calibrated scale to the nearest 0.1 kg, and WC was assessed at the umbilicus using a Gullick 2 flexible tape measure to the nearest 0.1 cm. BMI was calculated as weight in kg/height in M2. A venous blood sample was collected to assess HbA1c at baseline and 3 months, using HPLC on a D-10TM analyzer (Bio-Rad Laboratories Inc., Hercules, CA).

Questionnaires. Participants completed the following questionnaires: (1) the Diabetes Self-Care Activities Measure (SDSCA), which consisted of 12 items that ask about different areas of self-care for persons living with T2D, such as diet, physical activity, medication, glycemic screening, and smoking. Responses can be graded from 0 to 7 depending on the number of days (within the previous week) that each activity has been performed by the subject. Except for smoking, which can only attribute one point. A lower punctuation represents less commitment to favorable self-care activities for diabetes control (19); (2) the diabetes distress scale (DDS) to capture personal stress, interpersonal stress, or stress related to medical care or treatment, which is composed of 17 items related to personal stress, interpersonal stress, or stress related to their medical care or to their treatment, where each item can be responded to on a Likert-type scale where 1 represents no problem at all and 6 represents a very severe problem (20); and (3) the diabetes knowledge questionnaire (DKQ-24) with a maximum score of 24 reflecting the best knowledge (21). All instruments have been validated previously in Spanish.

### 2.5 Statistical analysis

Descriptive analyses included mean and SD for variables with parametric distribution and median with interquartile range for variables with non-parametric distribution. Changes in clinical measures before and after the intervention were examined using a paired sample t-test. Analysis was performed with StataBE 17.

## 3 Results

### 3.1 Study feasibility

#### 3.1.1 Implementation of DSMES

We were able to design and implement a course to train CHWs; the course was developed in alignment with the self-management program of the American Diabetes Association and the Association of Diabetes Care and Education Specialists (22).

CHW recruitment was done in the community where the clinic is located, since cultural grounding of the curriculum was an important part of the intervention. Flyers were posted in convenience stores and churches, and clinic patients were asked if they had relatives that might be interested in participating. As a result, nine CHWs were enrolled in the training course, 2 of whom lived near the community where the study was performed, 2 had informal jobs in the same community, and the remaining 5 were relatives of patients or relatives of people living in the community. Among the group, four individuals had previous training in health sciences as health promoters or nurses. The training course for CHWs had a duration of 2.5 months via in-person sessions and aimed to train CHWs on relevant topics related to diabetes self-monitoring and care to prevent complications but also to promote leadership and communication skills in CHWs. The training course included workshops to expose CHWs to clinical settings and real-world scenarios for diabetic patients, fostering discussions with real patients, **Table 1** enlists the topics included in each session. CHWs that participated in the intervention were selected, once training was completed, some criteria included a result above 20 in the diabetes knowledge questionnaire (DKQ), desire and commitment to participate in the study (1.5-month time frame) with six in-person sessions, and those who completed the hiring protocol for our institution.

**Table 1:** Educational content of the DSMES delivered by CHWs to low-income adults with T2D.

Session	Topics
1	Introduction to the program, and what to expect. General concepts in diabetes, monitoring, healthy eating, and exercise. Discussion about expectations and personal experiences.
2	Types of medications, taking medications, and common cultural misconceptions about medication taking. Discussion about best practices for taking medications.
3	Self-monitoring blood glucose and relation to medication and food intake, and to exercise Practice with glucometers, and physical activation activity
4	Common complications in people living with diabetes and how to prevent them. Discussion about experiences with complications either self or from a family member.
5	How to talk to health care professionals about medications and complications Simulation activity about the interaction with PCPs.
6	Emotional support, menu planning, and where to get more information Stress management activity, and online-search for trusted resources.

#### 3.1.2 Implementation of SDM

The SDM curriculum was designed with the aim of providing primary care providers with the basic theoretical knowledge, skills, abilities, and resources necessary to facilitate SDM during a typical T2D clinical encounter. SDM seeks to establish an active discussion between PCPs and patients during consultations so that they can together select the best treatment option. To achieve this, PCPs offer information about the patient’s health condition and openly discuss available treatment options with the participant, as they are encouraged to actively participate by sharing personal information about their preferences, values, and contextual factors that could influence the decision-making process. This open, participatory environment seeks to improve participants’ understanding of the reasons behind therapeutic interventions related to their medical condition, as well as the pros and cons of the available therapeutic options. Overall, this information can aid in jointly deciding on the best course of action.

The SDM training consisted of a series of online, synchronous workshops led by a team of researchers (i.e., a clinical health psychologist and a physician) from Universidad Latina in Costa Rica. Training tools and resources were translated to Spanish when necessary, including materials from the SDM Toolkit (Train-the-Trainer Tools for Teaching SDM in the Classroom and Clinic) (23). The course was structured into four modules with a duration of one hour, delivered weekly, followed by two individual feedback/coaching sessions after reviewing and evaluating the PCPs own videotaped SDM consultations with T2D patients (permission was obtained to record the session) at the clinic. The evaluation was intended for three specific areas: (1) the amount and quality of the information provided by the PCP with respect to their condition in terms that the participant can understand; (2) the willingness of the PCP to provide different therapeutical interventions with a clear explanation of the advantages and disadvantages of each option; and (3) a discussion related to patients’ preferences and values, so that both can engage in a formal evaluation of the best option for treatment.

Each week, PCPs were provided with material to read and discuss during online sessions; relevant topics for discussion included medical models describing different approaches to the doctor-patient relationship and psychosocial factors related to diabetes medical outcomes, SDM skills, and techniques. Other activities included role-playing exercises using case studies. The PCP trainees were sent pre- and post-course evaluations via email to assess their general knowledge of SDM as well as their perceptions and feasibility for implementation.

#### 3.1.3 Participant screening and enrollment

**Figure 1:**
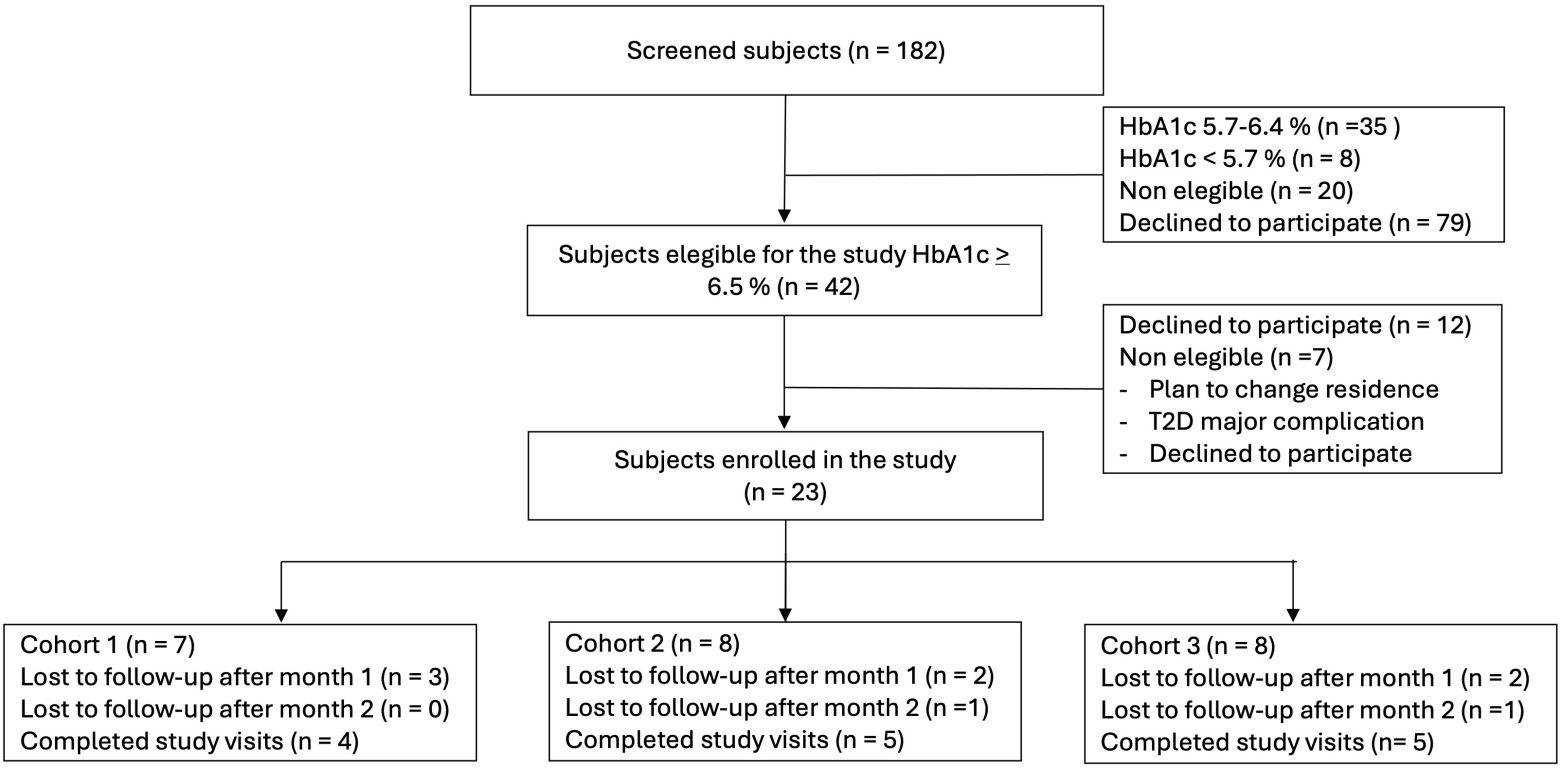
Study flow chart.

Over a 3-month period, 182 community residents were screened using POC testing of HbA1c. Of those, 42 had an HbA1c ≥ 6.5% and were referred to the clinic for follow-up, where 23 participants agreed to participate in the study (**Figure 1**).

### 3.2 Study acceptability

#### 3.2.1 Attendance and retention

Participants were followed for a total of 3 months. After enrollment participants were invited to a total of 6 weekly sessions with CHWs to address topics on DSMES. Sessions were scheduled at noon on three different weekdays; participants were able to select one of these days based on their availability. For each CHW, we constructed a cohort of up to eight study participants. Attendance to these sessions was reported in 80% of participants. During sessions, participants made weekly action plans, shared experiences, and helped each other to solve problems or issues related to their condition within the previous week. Furthermore, WhatsApp groups were created for each CHW, where constant communication was fostered with their respective cohorts of study participants. From the 23 enrolled participants in our study, 14 completed all three study visits.

#### 3.2.2 Study participant characteristics

Study participant characteristics at baseline are shown in **Table 2**; the mean age was 57 (±13.3) years, with 65% of participants being female.

**Table 2:** Baseline characteristics of enrolled study participants (*n* = 23).

Characteristic	Mean ± SD number (%) or mean (min–max)
Age (years)	57.7 ± 12.9
Sex M/F	8 (34.78)/15 (65.22)
Weight (kg)	77.5 ± 24.5
Height (mts)	1.57 ± 0.1
Body Mass Index (kg/m^2^)	30.9 ± 7.9
Waist circumference (cm)	96.2 ± 19.4
Hip circumference (cm)	106.4 ± 17.2
Waist-hip index	0.9 ± 0.1
Height-waist index	0.6 ± 0.1
HbA1c (%)	10.1 ± 2.7
Perceived stress	2.7 (1.1–4.9)
Diabetes knowledge	13.6 (9–19)
Diabetes self-care	3.2 (1.6–5.1)

### 3.3 Changes in clinical outcomes

To explore the potential clinical impact of the intervention, we examined changes in HbA1c %, anthropometric measurements, and psychosocial behaviors over time. HbA1c, weight, body mass index, hip circumference, waist to hip ratio, height to waist ratio, all decreased over the three-month period (**Table 3**). Similarly, diabetes knowledge and diabetes self-care behaviors increased compared to baseline.

**Table 3:** Changes in diabetes related study outcomes (n = 14).

Characteristic	Baseline	3 months
Weight (kg)	73.9 ± 16.7	70.4 ± 18.5
Body mass index (kg/m^2^)	29.7 ± 5.9	28.2 ± 6.6
Waist circumference (cm)	95.1 ± 20.9	91.2 ± 20.6
Hip circumference (cm)	106.4 ± 18.1	104.7 ± 19.6
Waist-hip index	0.89 ± 0.11	0.87 ± 0.09
Height-waist index	0.61 ± 0.13	0.59 ± 0.13
HbA1c (%)	10.1 ± 2.7	9.4 ± 3.1
Perceived stress	2.7 ± 1.3	1.9 ± 0.8
Diabetes knowledge	13.6 ± 3.2	15.5 ± 2.5
Self-care behaviors	3.2 ± 1.2	4.2 ± 0.9

## 4 Discussion

This pilot study demonstrated the feasibility and acceptability of a community-based program to screen, refer, and provide care to low-income individuals living with T2D residing in a low-income community in Jalisco, Mexico. Furthermore, individuals who participated in the intervention and returned for follow-up testing exhibited promising changes in multiple clinical indicators of T2D-related health outcomes.

As part of the pilot study, several lessons were learned that may inform the expansion of this program to other communities. Importantly, we were able to reach a higher number of residents, using a mobile unit and performing POC HbA1c screening. These individuals represent participants who do not regularly seek care and would likely benefit from increased access to T2D resources. Nevertheless, the majority of those screened were not interested in enrolling in the study.

We learned that interpersonal abilities, such as good communication, as well as knowledge related to the disease, are necessary for people living with T2D to engage in a meaningful discussion about treatment options during SDM. This is challenging in communities where access to education might be limited and health literacy is low. This notion was reinforced by the PCPs in the study, as they acknowledged many challenges during SDM among participants who were hesitant to engage in discussions about treatment options during PCP visits.

With respect to previous studies where DSMES has been used as an intervention in low-income people living with T2D, the study performed by Whittemore et al. (24) reported an 89% attendance of participants to sessions and 6.4% attrition. In our study rates of participation and attrition were 80% and 39%, respectively, with most of the no-shows occurring after the first month. This may be attributed to the lack of ongoing DSMES group activities after our 6-week duration of the DSMES course.

In the case of DSMES as an intervention, larger studies have shown that continued weekly group sessions could be a critical strategy in sustaining HbA1c improvements at 12- and 18-month follow-up (25). It would be ideal to find mechanisms to support patients in continuing their interaction with CHWs, with electronic devices and the use of tools or apps with gamification being a possibility to be further exploited, or having sessions every other week to extend the time of personal interaction within group activities.

Several studies have demonstrated the benefits of DSMES on glycemic control (26), and we observed that HbA1c was 0.68 units lower after the 3-month intervention period. However, these results should be taken with caution, as pilot studies often yield unstable or imprecise effect size estimates, particularly with small sample sizes. This may be the case in our study. Nevertheless, the potential clinical impact of a change in HbA1c coupled with other clinically relevant measures suggests that the intervention shows promise and warrants further investigation.

Furthermore, we observed promising increases in diabetes knowledge, which has been highlighted as a desirable outcome in people living with T2D (17). With respect to the contribution of SDM to outcomes observed in our study, we conducted interviews with PCPs to understand the level of participation and engagement; although participants were attending the consultations, it was difficult to engage them in discussions around their treatment, as they were more interested in the PCPs’ opinion rather than having to create their own about their treatment. Previous studies have shown that the use of SDM can be favorable in reducing HbA1c (27), but in our experience, in order for people living with T2D to discuss self-management strategies for healthy dietary habits, exercise promotion, or decisions related to screening or treatment, they need to be fully informed, something that can be achieved by first delivering a complete DSMES program through CHWs. Most of the literature around SDM is focused on the intervention itself rather than measuring outcomes, and specifically there is scarce information about the effects of SDM on long-term health (28, 29). It would be desirable to conduct future studies with the purpose of measuring the effect of DSMES on SDM itself, since the former may have a beneficial effect on the latter.

Finally, it is of the upmost importance that the DSMES be culturally grounded by CHWs, as CHWs are aware of the real-life context experienced by people living with T2D. For example, CHWs are often able to appreciate the day-by-day challenges related to food insecurities, access to spaces for physical activity or equipment, treatment options, medical care, transportation, job insecurities, educational context, stress management, and decision-making. Therefore, some consideration needs to be made with respect to CHW training to include the development of rapport, cultural competence, and communication skills to deliver health promotion programs and at the same time be sensitive to community members’ needs.

Our findings support community-based screening for T2D in communities affected by SDoH. In addition, the intervention combining culturally grounded DSMES delivered by CHWs with PCPs employing SDM showed promise for improving important clinical T2D health indicators, including HbA1c and diabetes knowledge. Despite this, some limitations are worthy of comment. These include the small sample size, non-randomized design, high attrition rates, and focus on a single community. Future studies that include more rigorous designs, larger sample sizes, and more comprehensive measures of SDoH are warranted.

## 5 Conclusions

In conclusion, we observed that a coordinated approach to identify and refer low-income adults with T2D for DSMES and SDM was feasible and acceptable among a community impacted by SDoH.

## Data Availability

The raw data supporting the conclusions of this article will be made available by the authors, without undue reservation.
